# Estimation of the probability risks of African swine fever outbreaks using the maximum entropy method in North Sumatra Province, Indonesia

**DOI:** 10.14202/vetworld.2022.1814-1820

**Published:** 2022-07-26

**Authors:** Roza Azizah Primatika, Etih Sudarnika, Bambang Sumiarto, Chaerul Basri

**Affiliations:** 1 Department of Veterinary Public Health, Faculty of Veterinary Medicine, Universitas Gadjah Mada, Yogyakarta, Indonesia; 2 Veterinary Public Health Study Program, Faculty of Veterinary Medicine, IPB University, Bogor, Indonesia; 3 Department of Animal Disease and Veterinary Public Health, Faculty of Veterinary Medicine, Institut Pertanian Bogor, Bogor, Indonesia

**Keywords:** African swine fever, environmental niche models, maximum entropy, probability risk

## Abstract

**Background and Aim:**

African swine fever (ASF) is an infectious disease and a major viral pig disease that threatens pork production in several locations globally. The mortality rate of ASF in domestic pigs is very high, causing a decrease in pig populations and significant economic losses for farmers. Environmental or ecological risk factors are the most important associated with the spread of the ASF virus. Environmental (or ecological) niche models are commonly used to estimate the probability of an event using the maximum entropy (Maxent) method. This study aimed to estimate the probability risk of future ASF outbreaks in North Sumatra, Indonesia.

**Materials and Methods:**

Secondary data from the National Animal Health System Database (iSIKHNAS), including data on the ASF outbreaks of 2019–2020 in North Sumatra, Indonesia, were used in this study. The first analysis performed involved the identification of environmental risk factors using multiple regression analysis. The second analysis performed was the estimation of probability risk for future ASF outbreaks in North Sumatra, Indonesia, using the Maxent method. Data processing was performed using Microsoft Excel, ArcGIS version 10.5 software (ESRI, California, United States), Maxent version 3.4.4 software, and Rstudio (http://www.r-project.org/).

**Results:**

The Maxent method was found to be highly accurate with a statistically significant area under the curve value of 0.860. The greatest contributing environmental factor identified by the model was the harbor, which contributed 57%. The range of high probability risk of future ASF outbreaks was found to be 0.723–0.84.

**Conclusion:**

The estimation of the highest probability risk of future ASF outbreaks in North Sumatra, Indonesia, was 0.723–0.84. The most contributing environmental factor identified using the Maxent method was harbors, at 57%. This methodology can be used to carry out subsequent ASF analyses and contribute to developing prevention and control strategies in this area.

## Introduction

African swine fever (ASF) is an infectious viral pig disease that has become a severe problem affecting global pork production in several locations [1, 2]. ASF is caused by the African swine fever virus (ASFV), a Deoxyribonucleic acid arbovirus belonging to the Asfarviridae family [3]. ASF can attack both domestic pigs and wild boars. The pig industry is an important source of animal protein in the worldwide production of livestock [4]. In North Sumatra, Indonesia, consumption of protein per capita is 3.65 g/day on average, of which almost 55% is sourced from pork [5].

The first outbreak of ASF in Indonesia occurred in North Sumatra in August 2019 and was detected by the animal disease investigation center. The total number of ASF cases in 2019 in the North Sumatra Province was 465 outbreaks among backyard swine [6]. The mortality rate of ASF in domestic pigs is very high, causing a decrease in the pig population and significant economic losses for farmers [7, 8]. ASFV had spread to domestic pig populations in all districts of North Sumatra by 2019–2020. There is currently no vaccine or cure for ASF, so early detection, laboratory diagnosis, and strict biosecurity measures are the primary control methods to deal with ASF outbreaks in the field [9]. Contact with diseased animals and insects, ingestion of contaminated pig products, and tick bites are all common ways for ASFV to spread [10]. In Eurasia, a region that is also fighting ASF, limiting the spread of disease in pig farms is very difficult where free-living wild boars remain as vectors and reservoirs of the virus. The probability of disease transmission among livestock facilities originating in wildlife vectors is determined by a combination of ecological, demographic, and behavioral factors [11].

Environment or ecological factors are the most important types of factors impacting the spread of ASFV in North Sumatra. These environmental factors include transportation, rivers, landfills, and roads. Environmental (or ecological) niche models (ENMs) are strategic tools that combine occurrence and environmental data to create a correlative model of the environmental variables that match a disease’s ecological requirements. This allows for the prediction of the incidence of disease cases in a selected area, in this case, ASF [12]. ENMs can estimate the probability of future ASF outbreaks in North Sumatra using the maximum entropy (Maxent) method. The estimated probability risk of ASF can then be used to carry out subsequent ASF prevention and control strategies. The Maxent method estimates the probability risk of ASF using current data to estimate a set of functions relating to environmental variables and geographical potential. The probability risk of ASF in Indonesia has not been previously reported based on environmental factors, especially in North Sumatra.

Therefore, this study aimed to estimate the probability risk of future ASF outbreaks in North Sumatra, Indonesia, using the Maxent method.

## Materials and Methods

### Ethical approval

This study did not require ethical approval as it was conducted solely using available data.

### Study period and location

**Figure-1:**
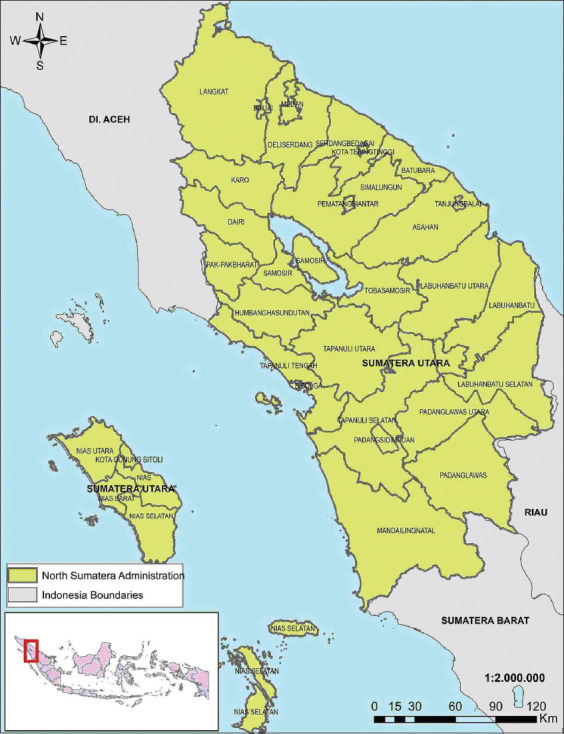
Map of North Sumatra Province, Indonesia [13].

The study was conducted from March 2021 to September 2021. This study was focused on the region of North Sumatra, Indonesia, located at 10–40 latitude, and 980–1000 longitude (Figure-1) [13]. The land area of North Sumatra Province is 72,981.23 km^2^ and is divided into 33 districts/cities. The total population in North Sumatra Province is 15,046,140 people, and the pig population in 2019 was 1,027,568 [5].

### Data source

The data used in this study consisted of secondary data obtained from the national animal health system database (iSIKHNAS) (http://wiki.isikhnas.com/) which was sourced from animal health officers of the Ministry of Agriculture of the Republic of Indonesia, animal health officers from the Department of Food Security and Livestock of North Sumatra Province, the Animal Disease Investigation Center of Medan, and the Bureau of Statistics. All reported ASF outbreak data used in this study occurred in 2019 and 2020. The secondary data utilized included the dates the outbreaks occurred, the locations of the outbreaks (district, sub-district, and village), the number of pig deaths resulting from ASF, local pig population, local human population, and geographical and infrastructure data on roads, rivers, landfills, railway stations, airports, and harbors. The data were processed using Microsoft Excel (Microsoft Corporation, Washington, United States), ArcGIS 10.5 software (ESRI, California, United States), Maxent software version 3.4.4 (https://biodiversityinformatics.amnh.org/open_source/maxent/), and Rstudio (http://www.r-project.org/).

### Data collection

Secondary data were collected in each study location in North Sumatra Province. The coordinates of the outbreak locations in each district were identified using Google Maps (https://maps.google.com). Raster and vector data were plotted into a shapefile (*.shp*) with points in the 1984 World Geodetic System coordinate system. The information on roads, rivers, landfills, railway station, airport, harbor, and the base map used in this study were sourced from a map of the province of North Sumatra obtained from the Geospatial Indonesia portal (https://tanahair.indonesia.go.id). ASF outbreak coordinates and the number of swine deaths associated with each outbreak were entered into the geodatabase. In addition, pig and human population data from each district were obtained from the veterinary service authorities and the Bureau of Statistics (www.bps.go.id).

### Statistical analysis

Statistical analyses were carried out in two steps. The first step involved multiple linear regression analyses to examine environmental risk factors. The dependent variable was the density of ASF case outbreaks, and the independent variables were the density of roads (main and secondary roads), the density of rivers, density of railway stations, density of airports, density of harbors, the density of landfills, pig population density, and human population density. The density of the variables used kernel density spatial tools to calculate a measurement for point or polyline patterns per unit area of each environmental factor. Values were then extracted to indicate values for all kernel smoothed density surfaces [14]. Kernel density analyses were conducted using the spatial analyst toolbox in ArcGIS version 10.5 (ESRI), and the multiple linear regression analysis model was constructed using Rstudio (http://www.r-project.org/).

The second analysis estimated the probability of risk of future ASF outbreaks in North Sumatra using the Maxent method [15], which was used to create probability risk figures of future ASF outbreaks based on previously significant environment variables generated from multiple regression analyses. From large experimental data sets, potential species distribution data, and predicted optimal habitat areas, the maximum Maxent method has been most extensively applied to develop descriptive and predictive models of biological systems, especially complex biological networks [16, 17]. The area under a receiver operating characteristic curve (AUC) produced using model predictions can be used to measure the model’s predictive accuracy [18, 19]. From 2019 to 2021, the locations of ASF outbreaks in domestic swine in North Sumatra were treated as known outbreaks (presence data).

The Maxent method was analyzed using Maxent software version 3.4.4 [18, 20]. The relative contribution of each variable to the estimated probability was determined using Jackknife statistics. Explanatory variables that contributed <1% to the model were eliminated. Data processing in the Maxent method produces means, as well as 95% confidence intervals for response curves which describe the association between each environmental parameter, as well as the probability of each grid cell being ASF positive. Monte Carlo simulations were also conducted, and 100 replications were performed. The model was trained by randomly selecting half of the points and then running them on the remaining data. The raster surface produced by the Maxent method approximated the median probability distribution of ASF outbreaks in the study area [19].

## Results

The results of the multiple linear regression analyses with p < 0.05, and which were identified as significant risk factors, were the pig population density, road density, harbor density, railway station density, river density, and landfill density. Based on regression analyses, the probability risk estimation was analyzed using the Maxent method.

**Figure-2:**
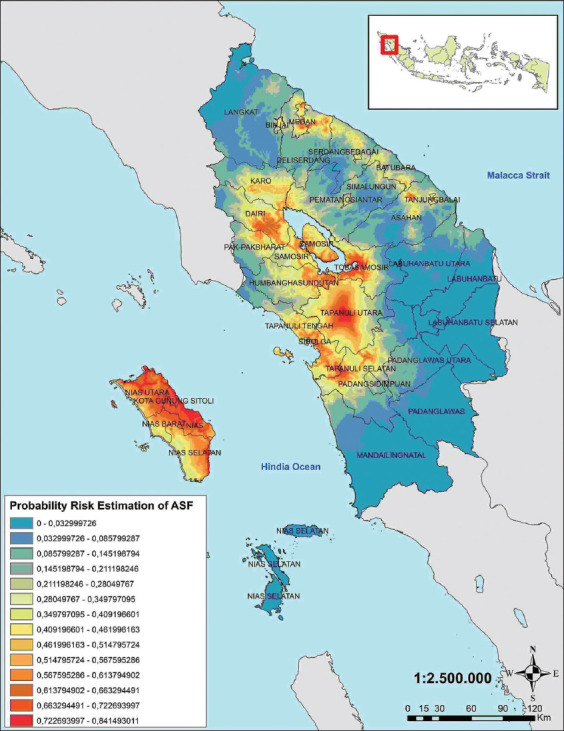
The probability risk estimation map of future African swine fever outbreaks in North Sumatra, Indonesia [Source: ArcGIS software version 10.5 (ESRI, USA)].

**Figure-3:**
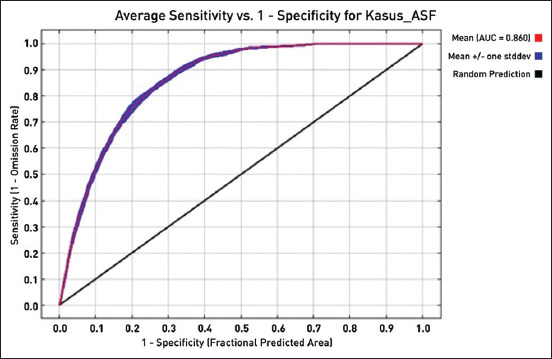
Area under the curve value of the receiver operating characteristic curve of the African swine fever distribution model.

**Figure-4:**
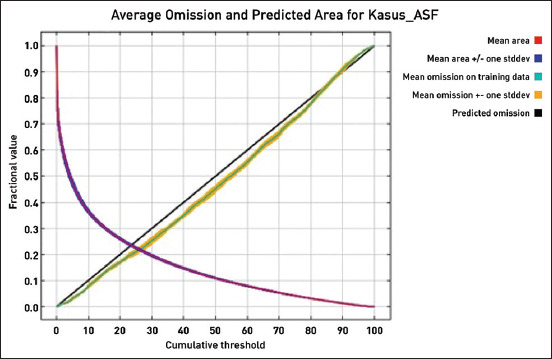
Average omission and predicted area curve for African swine fever cases in North Sumatra, Indonesia.

**Figure-5:**
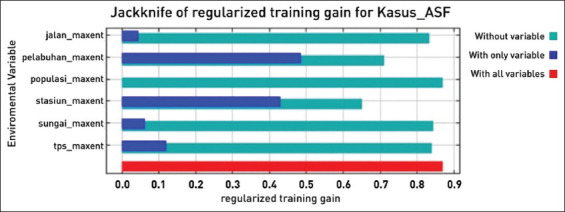
The Jackknife test for evaluating the relative importance of environmental variables for African swine fever outbreaks in North Sumatra, Indonesia.

The environmental variables selected for the study in the multiple regression analysis were uploaded to the Maxent software to estimate the probability risk of future ASF outbreaks throughout North Sumatra, Indonesia (Figure-2). The highest probability was marked in red on the map, which had a probability of 0.723–0.841. The Maxent model for the future ASF outbreak provided satisfactory results, with an AUC value of 0.860, higher than the 0.5 of a random model (Figure-3). In addition, the AUC values were supported by omission averages and predicted areas for ASF cases (Figure-4). Omission averages and predicted areas showed that omission rates (on training tests and test samples) approached the predicted omission line. The closer the two lines were to the predicted omission, the more valid the displayed data. The location of harbors was found to be the factor that contributed most to the model (57%), followed by railway stations (32.4%), roads (4.2%), landfills or final garbage dumps (3.9%), rivers (2.9%), and finally pig population (0%) (Figure-5 and Table-1).

**Table 1:** Percent contribution of environmental factors that influence the African swine fever outbreak risk estimation model using the Maxent method.

Variables	Percent contribution
Harbor	57
Railway station	32.4
Roads	4.2
Final garbage dump	3.6
Rivers	2.9
Pig population	0

## Discussion

As a result of the North Sumatra ASF outbreak of 2019–2020, researchers have developed techniques to prevent and control ASF. These techniques include the prediction of distribution areas of future ASF outbreaks based on several environmental variables [19, 21]. In this study, the future distribution areas for ASF outbreaks in North Sumatra Province were modeled using the popular Maxent software based on the Maxent method [16].

The model’s accuracy was measured using the value of the AUC test, which indicated that the model was very effective in measuring the presence or absence of ASF outbreaks in future periods [22]. This study’s results of ENMs using the Maxent method were characterized by excellent AUC values. The high AUC results confirmed that the Maxent method could effectively estimate the probability risk of ASF in North Sumatra.

An environmental variable contribution factor identified by the Maxent method was a harbor in North Sumatra Province. Harbors were, and remain, one of the primary transportation methods used in the movement of pigs in Indonesia. The presence of the harbor was found to be the most important factor in the spread of ASFV. In Indonesia, the movement of pigs is carried out by sea transportation from one island to another. Based on the information provided by the animal quarantine center in Belawan Harbor in North Sumatra Province, the inter-island pig trade is carried out by ship (personal communication). The harbor is also a strategic site for the transportation, buying, and selling of domestic pigs. Although contaminated transport trucks are a risk factor and a source of ASF transmission between farms, reports are not well documented [23]. In contrast, one of the causes of the spread of ASFV is food waste which is used by farmers as a swill for feeding pigs [1]. Food waste from overseas ships containing ASFV-contaminated pigs can also spread ASFV if farmers use this swill for feeding their pigs. In a study conducted in Tanjung Priok Harbor, ASFV from food waste containing pork had an ASFV prevalence of 8.69% detected in ships from China and the Philippines [24]. Port waste management has been conducted according to Marine Pollution 73/78 (MARPOL 73/78) standards concerning the prevention of pollution from ships, but in the port of Tanjung Priok, the availability of a Standard Operational Procedure to prevent the spread of ASFV remains to be developed by PT Pelindo II (Indonesia Port Corporation) for ship waste management [25].

The highest probability risk of future ASF outbreaks which have been red-marked is 0.723–0.841. Many factors influence the high risk of ASF in North Sumatra. In the model used in this study, the environmental factor that most strongly influenced the ASF risk probability was the harbor. On the map, we show that most red-markings are located on Nias Island, which has a high consumption of pork associated with its use in traditional ceremonies. The butchers there have to source pork from other areas, such as Jambi and Riau Provinces, because of the very high consumer demand. In addition, wild boars are frequently hunted and sold in this area of the province. A study conducted in Poland found ASFV in their wild boar population [26]. In addition, countries with a pig farming sector have the risk of transmitting ASF to other countries through food leftovers from planes, ships, or meat carried by individual travelers [4]. The risk of ASFV entry into the European Union (EU) is through three types of transport routes: returning trucks, trash from international ships, and waste from international flights. These will be referred to as transport-associated routes (TAR) here, and the study found that TAR in the EU was minimal [27].

On the other hand, an ASF study in Ontario stated that areas with high pig population densities, such as on pig farms, posed a high risk and could affect many farms [11]. The interaction of ecological, demographic, and behavioral variables both spatially and temporally is very complex in the spread of ASF [11, 28, 29]. To account for these interactions, a statistical framework based on random forest methods was established in Romania to examine the spatiotemporal aspects of the epidemics and their interactions with environmental, human, and agricultural factors [30].

The ideal control option for an ASF epidemic in industrialized pig herds with no contact with wild boars was projected to involve testing dead animals in protection and surveillance zones [31]. Many countries and regions have strategies to prevent ASF introduction, such as Africa, the EU, the United Kingdom, Romania, and Sardinia. The ASF prevention strategies applied in these examples are strict biosecurity measures on farms which increase the capacity of veterinary services to detect disease, to perform diagnosis, surveillance, management, and emergency and contingency response and care; thus strengthening the capacity of farmers to raise healthy pigs by increasing access to information and training. These programs also limit transportation in order to limit the trade movement of pork, pork products, and related human activities [23, 32, 33]. Indonesia’s geographical environment conditions and characteristics are different from other countries. An estimation of the probability risk of ASF in Indonesia is very useful to help develop government policies to prevent ASF in high probability risk areas through appropriate data-based control and biosecurity strategies.

## Conclusion

The estimation of the highest probability risk of future ASF outbreaks in North Sumatra was 0.723–0.841. The greatest contributing environmental factor using the Maxent method was a harbor, at 57%. Veterinary services must intensively control animal traffic and transportation to limit the trade movement of pigs and pork products. These efforts can be used to carry out subsequent ASF prevention and control strategies in North Sumatra.

## Authors’ Contributions

RAP: Designed the study, data collection and analysis, and drafted the manuscript. ES, BS, and CB: Designed the study, analyzed the data, and contributed to manuscript preparation. All authors have read and approved the final manuscript.
